# Unbiased analysis of mouse brain endothelial networks from two- or three-dimensional fluorescence images

**DOI:** 10.1117/1.NPh.9.3.031916

**Published:** 2022-05-18

**Authors:** Moises Freitas-Andrade, Cesar H. Comin, Matheus Viana da Silva, Luciano da F. Costa, Baptiste Lacoste

**Affiliations:** a The Ottawa Hospital Research Institute, Neuroscience Program, Ottawa, Ontario, Canada; b Federal University of São Carlos, Department of Computer Science, São Carlos, Brazil; c University of São Paulo, São Carlos Institute of Physics, FCM-USP, São Paulo, Brazil; d University of Ottawa, Faculty of Medicine, Department of Cellular and Molecular Medicine, Ottawa, Ontario, Canada; e University of Ottawa Brain and Mind Research Institute, Ottawa, Ontario, Canada

**Keywords:** endothelium, cerebrovascular, mouse brain, angiogenesis, image analysis, computation, unbiased, networks, connectivity

## Abstract

**Significance:**

A growing body of research supports the significant role of cerebrovascular abnormalities in neurological disorders. As these insights develop, standardized tools for unbiased and high-throughput quantification of cerebrovascular structure are needed.

**Aim:**

We provide a detailed protocol for performing immunofluorescent labeling of mouse brain vessels, using thin (25  μm) or thick (50 to 150  μm) tissue sections, followed respectively by two- or three-dimensional (2D or 3D) unbiased quantification of vessel density, branching, and tortuosity using digital image processing algorithms.

**Approach:**

Mouse brain sections were immunofluorescently labeled using a highly selective antibody raised against mouse Cluster of Differentiation-31 (CD31), and 2D or 3D microscopy images of the mouse brain vasculature were obtained using optical sectioning. An open-source toolbox, called Pyvane, was developed for analyzing the imaged vascular networks. The toolbox can be used to identify the vasculature, generate the medial axes of blood vessels, represent the vascular network as a graph, and calculate relevant measurements regarding vascular morphology.

**Results:**

Using Pyvane, vascular parameters such as endothelial network density, number of branching points, and tortuosity are quantified from 2D and 3D immunofluorescence micrographs.

**Conclusions:**

The steps described in this protocol are simple to follow and allow for reproducible and unbiased analysis of mouse brain vascular structure. Such a procedure can be applied to the broader field of vascular biology.

## 1 Introduction

The brain, with its elevated energy consumption and limited energy storage, is highly dependent on a steady supply of nutrients carried by blood vessels. In addition to the need for a dense vasculature, the brain also requires a controlled environment providing suitable conditions for healthy neurotransmission. Proper brain maturation, function, and aging are supported by (1) the establishment and maintenance of endothelial networks (neovascularization and angiogenesis) for efficient perfusion, (2) the formation and integrity of the blood–brain barrier to uphold brain homeostasis, and (3) the regulation of cerebral blood flow to match energy demands. The anatomical substrate of these features is known as the neurovascular unit, a multicellular structure in which endothelial cells play critical roles in regulating brain health through structural and functional interactions with neurons, pericytes, astrocytes, and microglia.^1^^–^^4^

Although knowledge on the relationships between blood vessels and neural networks is getting traction, a neurocentric approach to neurological disorders has generally led to limited understanding of cerebrovascular remodeling in brain maturation and diseases. However, growing evidence supports the contribution of endothelial defects to the onset and/or progression of neurological disorders, including, but not limited to, Alzheimer’s disease, multiple sclerosis, and autism spectrum disorders.^5^^–^^11^ As such, there is an urgent need to implement open-source and standardized methods for systematic and high-throughput analysis of the cerebrovascular structure in laboratory models.

We propose a simple, reliable, and inexpensive protocol aimed at immunostaining mouse brain endothelial networks on fixed tissues, followed by fluorescence of optical sectioning to process two- or three-dimensional (2D or 3D) digital images using computerized methods. This protocol provides a means to unbiasedly quantify important metrics of cerebrovascular structure.

## 2 Methods

### 2.1 Histology for 2D Vascular Imaging

#### 2.1.1 Fixation of brain tissue samples for 2D vascular imaging

*Note:* For this protocol, mice are not perfused, for we found that perfusion, including with fixatives, affects the quality of CD31 immunostaining.

1. Euthanize mice by cervical dislocation.
2. After euthanasia is confirmed, decapitate mouse and quickly extract the brain.
3. Place the brain in cold 50 mM phosphate-buffered saline (PBS) pH 7.4 (Table 1), and remove cerebellum as well as olfactory bulbs.
4. Immerse the brain in a solution of 4% paraformaldehyde (PFA) (Electron Microscopy Sciences Cat #15713-S) in PBS overnight at 4°C.
5. After overnight fixation, rinse sample in PBS, and place cortex in a 30% sucrose/PBS solution at 4°C for cryopreservation. The cortex must remain in the sucrose solution at 4°C until it completely sinks to the bottom of the tube (or plate well).
6. Embed the cryoprotected brain in optimal cutting temperature (OCT) compound on dry ice within a molding receptacle.
7. Store embedded brains at −80°C (up to 1 year for high quality immunofluorescence).

**Table 1:** PBS (50 mM, pH 7.4) 1× buffer.

Reagent	Amount
${\mathrm{Na}}_{2}{\mathrm{HPO}}_{4}$	5.87 g
${\mathrm{NaH}}_{2}{\mathrm{PO}}_{4}H_{2}O$	1.20 g
NaCl	9.00 g
Sterile water	Fill up to 1 L
Total	1 L

#### 2.1.2 Cryosectioning for 2D vascular imaging

1. Set the cryostat temperature to at least −20°C. The quality of sections is sensitive to the cryostat temperature, which also depends on the ambient humidity.
2. Once the temperature in the cryostat is reached, place the brain sample in the cryostat chamber to allow the temperature of the sample to equilibrate for about 1 h.
3. Charged microscope slides must be used and labelled appropriately wearing gloves to avoid fingerprints on the charged surface.
4. Keep slides at room temperature for at least an hour, so the section can thoroughly dry and adhere to the glass surface.
5. Set section thickness at 25  μm and cut at a slow and constant speed.
6. Store slides with mounted sections at −80°C. We found that the quality of the sections begins to deteriorate after six months of storage. However, this is highly dependent on the number of freeze/thaw cycles to which the sections are subjected.

#### 2.1.3 Endothelium immunostaining on 25  μm-thick brain sections for 2D vascular imaging (i.e., immunofluorescent staining on slides)

##### Day 1

**Fig. 1:**
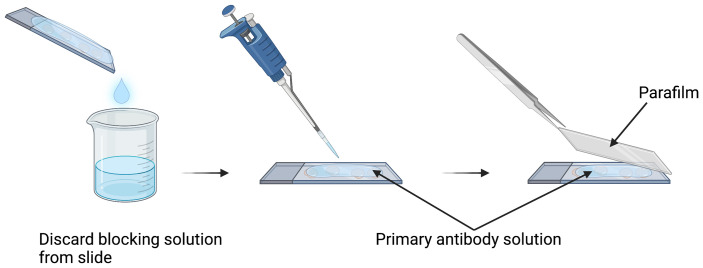
Primary antibody incubation on slides. Image depicts slide incubated with a small volume (200  μl) of primary antibody solution covered with a small piece of parafilm. Parafilm is large enough to cover all of the sections but slightly smaller than the width of the microscope slide. This method allows for the use of small volumes of antibody solution without the possibility of evaporation.

1. Bring microscope slides with cryo-sections to room temperature (RT) for 20 min.
2. Fill three slide containers with 50 mM PBS.
3. Rinse slides three times for 5 min in PBS.
4. Fill two slide racks with permeabilizing solution: PBS with 0.5% Triton^®^ X-100 (0.5% PBT) (Table 2).
5. Rinse slides twice for 5 min in 0.5% PBT.
6. Before or during steps 3 to 5, prepare enough blocking solution for the experiment (Table 3).
7. Add 600  μL of blocking solution onto each slide, and incubate for 1.5 h at RT in a humid slide chamber.
8. Dilute the primary antibody in blocking solution at the chosen concentration. Antibody: rat anti-mouse CD31 (BD Pharmigen, Cat #553370) at 1:200 dilution.
9. Discard blocking solution from the slide, replace with 200  μL of primary antibody solution, and delicately cover with parafilm (Fig. 1). Incubate overnight at 4°C in a humid slide chamber.

**Table 2:** 0.5% PBT buffer.

Reagent	Final concentration	Amount
TritonX-100	0.5%	5 mL
1× PBS	n/a	995 mL
Total	n/a	1 L

**Table 3:** Blocking solution.

Reagent	Final concentration	Amount (mL)
Normal donkey serum	10%	5
10% cold water fish skin gelatin	0.5%	2.5
0.5% PBT	n/a	42
Total	n/a	50

*Note*: The serum helps prevent non-specific binding of the secondary antibody. The serum used should correspond to the species of the secondary antibody host. The fish gelatin helps prevent non-specific binding of the primary antibody.

##### Day 2

10. Discard primary antibody solution.
11. Rinse slides three times for 5 min in fresh 0.5% PBT.
12. Prepare dilution of secondary antibody in blocking solution: Donkey anti-Rat AlexaFluor 488 (Fisher Scientific, Cat # A21208) at 1:300 dilution.
13. Add 600  μL of secondary antibody dilution onto each slide. Incubate for 2 h at RT in a humid slide chamber protected from light.
14. Rinse slides three times for 5 min in 0.5% PBT.
15. Rinse slides two times for 5 min in 0.1M PB (Table 4).
16. Quickly dip slides in distilled water three times to remove excess phosphate.
17. Add mounting Fluoromount-G medium (Electron Microscopy Sciences Cat # 17984-25) or ProLongGold with DAPI (Thermo Fisher Scinetific Cat#. P36935) onto each slide, and carefully place a coverslip. Leave on bench (covered) to dry for 1 h at RT, and then store slides in a slide book at 4°C overnight for continued drying before imaging.

**Table 4:** PB (0.1 M, pH 7.4) 1× buffer.

Reagent	Amount
${\mathrm{Na}}_{2}{\mathrm{HPO}}_{4}$	11.74 g
${\mathrm{NaH}}_{2}{\mathrm{PO}}_{4}H_{2}O$	2.40 g
Sterile water	Fill up to 1 L
Total	1 L

### 2.2 Histology for 3D Vascular Imaging

*Note:* For this protocol mice are not perfused, as we found that perfusion, including with fixatives, affects quality of the CD31 immunostaining.

#### 2.2.1 Fixation of brain tissue samples for 3D vascular imaging

**Fig. 2:**
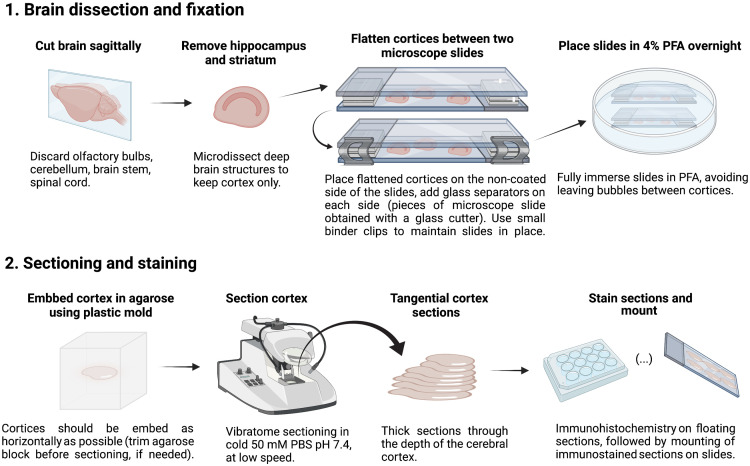
Procedure for preparing mouse brain for vibratome sectioning. Brains are cut in half along a sagittal line. The olfactory bulbs and cerebellum are removed. The cortex is placed in PBS with the striatum facing up, and then the striatum along with the hippocampus is removed. The cortex is then placed on the non-coated side of the slide with the striatum side of the cortex facing down (step 1). Two glass separators, which were obtained by cutting a microscope slide into small pieces, are placed on either side of the slide and a second microscope slide covers the brains as shown in the figure above (step 1). Cortexes are placed in 4% PFA overnight in a plastic cell culture dish. The following day, the cortexes are embedded in agarose (step 2). The embedded cortex is then sectioned tangentially with a vibratome (step 2), stained, and mounted (see also Refs. 5 and 12).

*Note*: We found that two glass separators are sufficient to flatten small brains from young mice (postnatal day 14) without distorting or damaging cortices. However, the number of glass separators may vary depending on the size of the brain.

1. Euthanize mice by cervical dislocation.
2. After euthanasia is confirmed, decapitate mice and quickly extract the brain.
3. Place the brain in cold 50 mM PBS pH 7.4 (Table 1), and remove cerebellum as well as olfactory bulbs.
4. Cut the brain sagittally and microdissect deep brain structures (striatum/hippocampus) to keep only the cortex (Fig. 2).
5. Flatten cortices between two microscope slides that are separated by glass separators, as illustrated in Fig. 2.

6. Fix brain tissue in 4% paraformaldehyde overnight at 4°C, and embed in agarose the following day (Fig. 2).
7. After fixation, rinse cortices three times for 5 min in PBS.
8. While rinsing brain tissue, prepare the agarose to embed the cortices within:
  • Weigh 3 g of agarose in a 250 mL Erlenmeyer flask.
  • Place in microwave after adding 100 mL of distilled water (∼1 to 2 min).
  • Add 15 mL of 10× PBS, mix, and microwave for another 30 s.
  • Add water to complete 150 mL total volume (resulting in 1× PBS/agarose solution).
  • Cool solution in 55°C water bath for 10 to 15 min while cortices continue to rinse in PBS.
9. Label the molds, and pour the agarose into the mold to fill it approximately halfway. Allow the agarose to slightly polymerize, and then place the flatten cortex on the polymerizing agarose. This will allow the cortex to stay afloat and not sink to the bottom of the mold while also keeping the cortex horizontal, which will facilitate vibratome sectioning later.
10. Pour in more agarose to fill the mold. Note: Do not wait too long between the first and second pour; otherwise, the block will split in two during vibratome sectioning.
11. A small spatula may be used to adjust the orientation of the cortex before agarose sets.
12. Place the mold with the cortex on ice for 5 min to quickly set the agarose.
13. Molds with the cortex can be stored in a Ziplock bag with a few drops of PBS (to prevent dryness) at 4°C. Cortices should be sectioned within two days, before bacterial or fungal contamination takes hold. Sections may be stored in a 12-well plate long-term at −20°C in “anti-freeze” solution (Table 5), prior to immunostaining. On the day of immunostaining, sections are rinsed with 50 mM PBS pH 7.4.

**Table 5:** Anti-freeze solution (store at 4°C).

Reagent	Amount (mL)
1× PBS	200
Glycerol	150
Ethylene glycol	150
Total	500

#### 2.2.2 Endothelium immunostaining of thick, free-floating sections for 3D imaging (i.e., from flattened cerebral cortex).

##### Day 1

Thick sections (50 to 150  μm) are obtained using a vibratome following microdissection of the cerebral cortex (see Fig. 2). Vibratome settings: Speed 4, Frequency 8 to 10 (Leica VT-1000S).

1. Using a small paint brush, transfer brain sections from the vibratome’s collecting receptacle into a 12-well plate filled with 50 mM PBS pH 7.4.
2. Blocking: gently remove PBS from wells using a transfer pipette and replace with blocking solution (Table 3). Incubate floating sections in blocking solution for 2 h at RT.
3. The primary antibody solution can be prepared during the blocking step. Dilute the primary antibody in blocking solution: rat anti-mouse CD31 (BD Pharmigen, Cat #553370) at 1:200 dilution. Keep at 4°C until ready for use.
4. After the blocking step, gently remove blocking solution from wells using a transfer pipette, and immediately add the primary antibody solution (avoid drying). Incubate overnight at RT under slow, gentle agitation for sections to move slowly in wells.

##### Day 2

5. Gently remove primary antibody solution using a transfer pipette, and rinse sections three times for 6 min in 0.5% PBT, under medium agitation.
6. Prepare dilution of secondary antibody in blocking solution: donkey anti-rat AlexaFluor 488 (Fisher Scientific, Cat # A21208) at 1:300 dilution.
7. Incubate sections with secondary antibody solution for 3 h at RT, protected from light, under gentle agitation.
8. Gently remove secondary antibody solution using a transfer pipette and rinse sections twice for 5 min in 0.5% PBT, under medium agitation.
9. Again, rinse sections twice for 5 min in 0.1M PB, under medium agitation.

##### Mount sections

10. Add a few drops of 0.1M PB onto slide to facilitate section placement.
11. Using a small soft paint brush, gently mount brain sections on the slide, and let sections dry for approximately 20 min at RT, covered from light (until sections become transparent).
12. Quickly dip slides in distilled water three times to remove excess phosphate.
13 Add mounting Fluoromount-G medium (Electron Microscopy Sciences Cat # 17984-25) onto each slide, and carefully place a coverslip. Leave on bench (covered) to dry for 1 h at RT, and then store slides in a slide book at 4°C overnight for continued drying before imaging.

### 2.3 Image Capture of Immunofluorescently Labeled Cortical Vessels from 25  μm-Thick Mouse Brain Sections for 2D Imaging

**Fig. 3:**
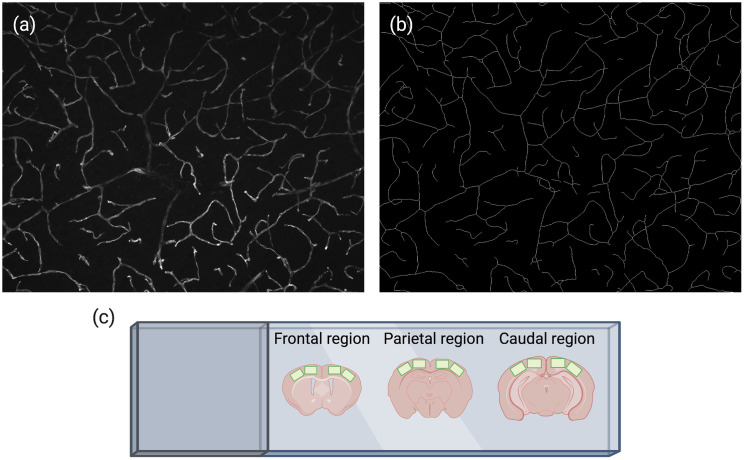
(a) Maximal intensity projection of CD31-stained vessels obtained by 10-μm-deep z-stacks. (b) Skeleton (see Sec. 3) representing the vasculature shown in (a) clearly captures all vessels within the 10  μm depth. (c) Illustration of cortex regions (green boxes) where images are captured for 2D analysis.

For images, immunostained sections were examined using a Zeiss Axio Imager M2 microscope equipped with a digital camera (Axiocam 506 mono) and the Zeiss ApoTome.2 module for optical sectioning. Alternatively, a confocal microscope can be used. For 2D imaging, ×20 objective (Zeiss; Plan-APOCHROMAT; 20x/0.8) was used to acquire 10-μm-deep z-stacks that were subsequently transformed into a maximal intensity projection image. Obtaining 10-μm-deep z-stacks (1  μm steps) allows for accurate quantification of vessels in one anatomical plane (Sec. 3) from maximal intensity projection images (Fig. 3).

### 2.4 Image Capture of Immunofluorescently Labeled Cortical Vessels from Thick Mouse Brain Sections for 3D Imaging

**Fig. 4:**
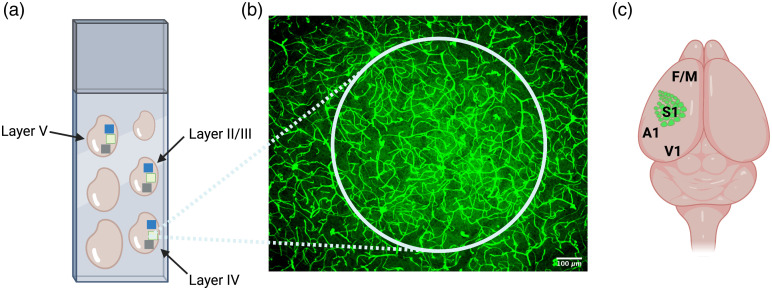
(a) The tangential serial cortexes on the slide illustrate the anterior (blue box), parietal (green box), and occipital (gray box) cortical regions that could be imaged. (b) By pushing exposure and brightness, layer IV of the primary somatosensory barrel cortex is easily visible via background immunofluorescence. Tangential serial sections above and below layer IV are considered as layer II/III and V, as indicated in (a). (c) The primary somatosensory barrel cortex (S1) can be used as a landmark to identify other neighboring brain regions, namely, frontal/motor cortex (F/M), auditory cortex (A1), and visual cortex (V1).

Using the Zeiss ApoTome with a ×10 magnification objective (Zeiss; Plan-APOCHROMAT; 10x/0.45), 60 to 70  μm-deep z-stacks (1  μm steps) can be acquired from thick sections for 3D reconstruction of brain vascular networks. With a confocal microscope, 90 to 100  μm z-stacks can be obtained. The three major subdivisions of the cerebral cortex (anterior, parietal, and occipital) can be imaged from the tangential sections [Fig. 4(a)]. By increasing exposure or brightness, it is possible to identify layer IV of the primary somatosensory barrel cortex from autofluorescence background in one of the serial sections [Fig. 4(b)]. Alternatively, vesicular glutamate transporter-2 (VGLUT2) immunostaining can be performed to image barrels. This specific cortical area can be used as a landmark to identify other cortical layers [Fig. 4(a)] and other important cortical regions [Fig. 4(c)], as previously described.^13^

## 3 Image Analysis and Quantifications

**Fig. 5:**
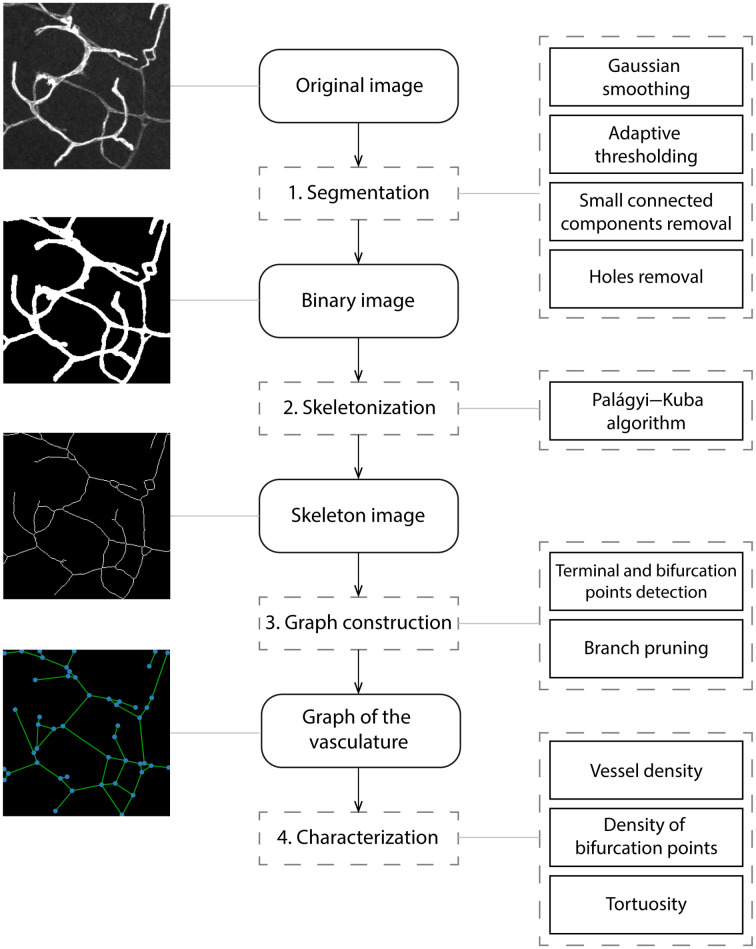
Illustration of the methodology used for characterizing vascular networks. The middle column shows the overall steps required for the analysis. The procedures used for implementing each step are shown on the right. Example images are shown on the left.

In the following sections, we describe automated procedures for characterizing digital vascular images using computerized methods. The general procedure involves four main steps: (1) the segmentation of the vascular network; (2) the identification of the skeleton of the blood vessels; (3) the definition of an appropriate representation of the vascular network, and (4) the calculation of relevant properties for characterizing the vasculature. The code implementing the described steps is available as a Python package called Pyvane and is available at https://github.com/chcomin/pyvane. In Pyvane, each of the aforementioned steps is implemented as a *processor* that can be freely changed using a custom code. The package also provides a set of default processors containing implemented algorithms that can be readily used. The default processors serve as a baseline protocol for analyzing vasculature networks. An illustration of the pipeline and the implemented algorithms is shown in Fig. 5.

Many recent approaches have been developed for segmenting and analyzing large blood vessel datasets.^14^^–^^19^ Such methodologies show excellent results even for large 3D volumes containing whole brain data. Still, there are two main advantages of Pyvane compared with the other methodologies. The first is that the default processors of Pyvane have been shown to provide good results for hundreds of images obtained from many different animals in previously published works.^5^^,^^13^^,^^20^^–^^22^ This contrasts with the recently developed approaches, which tested the algorithms on only three,^16^ five,^14^ nine,^19^ and fifteen^15^ microscopy images. Thus, it is expected that Pyvane can be easily adapted to new datasets. The second advantage is that, because the four main steps for processing blood vessel images have been built as modular *processors*, each of them can be easily adapted to new algorithms. For instance, the segmentation algorithm used in the segmentation processor can be readily changed to a convolutional neural network. This is also different from other approaches, which are usually distributed as a monolithic software to be used without changes.

### 3.1 Segmentation of the Vascular Network

For detecting the vascular network, we assume that blood vessels have a larger intensity than the background, that is, they are bright and the background is dark. We also assume that the image only contains blood vessels.

First, a Gaussian smoothing filter with a standard deviation of σ is applied. Typically, σ=1  μm. The smoothing is used for removing very high frequency variations in the image due to shot noise. Next, for 2D images, an adaptive thresholding operation is applied as follows. For each pixel i in the image, a weighted average intensity, Im(i), of pixels around pixel i is calculated. The weights are given by a Gaussian function centered on the pixel and having a standard deviation of R. The pixel is then classified according to the equation expressed as

Io(i)={1if  I(i)>Im(i)+C0if  I(i)≤Im(i)+C,

where I(i) is the intensity of pixel i in the original image, Io(i) is the respective intensity after the thresholding operation, and C is a threshold value that is typically 2 or 3. This operation amounts to classifying a pixel as belonging to a blood vessel if its intensity is larger than the average intensity of nearby pixels. A value of C>0 is useful for avoiding the classification of background regions as blood vessels. The whole procedure can be easily implemented by convolving the image with a Gaussian filter and then applying Eq. (1) for each pixel in the original and smoothed image.

Small connected components are removed because they are usually caused by shot noise, small fluctuations in sample illumination, or small tissues that are unrelated to blood vessels. Typically, components smaller than $50\;\mu m^{2}$ are removed. In a similar fashion, small holes are also removed from the image. They are associated with small regions inside blood vessels that end up being classified as background. Care must be taken to not remove actual background regions surrounded by blood vessels. Thus, holes with sizes of typically $10\;\mu m^{2}$ or less are removed.

For 3D stacks, the adaptive thresholding operation described above is applied to each Z-plane of the stack. This is useful for making the detection insensitive to changes in brightness along the depth of the sample. Small connected components, typically with a size of $350\;\mu m^{3}$ or less, are then removed. Because 3D stacks are unlikely to contain background regions completely surrounded by blood vessels, all holes are removed from the stack.

Since the CD31 staining is used for visualizing endothelial cells, blood vessels with large diameters may contain hollow regions. Still, the local thresholding method should provide good results if the radius used for the window is sufficiently large and if such hollow regions are brighter than the background surrounding the blood vessel, which was observed in our samples.

### 3.2 Skeleton Calculation

The result of the segmentation presented in the previous section is a binary image or volume containing the value 0 for background and 1 for the vasculature. It is important to transform the binary data into a more appropriate representation to facilitate the characterization of the vascular network. This is done by obtaining the skeleton, or medial axes, of the blood vessels. The skeleton is used for representing the vasculature as one pixel width lines for further processing. For 2D images, many popular algorithms can be used.^23^^,^^24^ For 3D stacks, the problem is more difficult because there is no unique definition of a 3D skeleton.^25^ We chose the Palágyi–Kuba algorithm^26^ due to its characteristic of keeping skeleton branches caused by small variations of the vessel walls. Such variations may be generated by vascular branches or may be a result of errors in the segmentation. Because the method keeps small skeleton branches, they can be further analyzed and pruned, if necessary. The pruning procedure is described below.

### 3.3 Representation as a Graph

The skeleton of the vasculature is further represented as a graph. Pixels belonging to the skeleton and having one neighbor pixel also belonging to the skeleton become terminal nodes. In a similar fashion, skeleton pixels having three or more neighbors in the skeleton become bifurcation nodes. Nodes are connected by an edge if there is a blood vessel segment between them. Furthermore, the path of the vascular segment represented by the edge is stored as an edge attribute. If multiple neighboring pixels are classified as a bifurcation, the respective nodes are merged, and a single node located at the average position of the merged nodes is created.

**Fig. 6:**
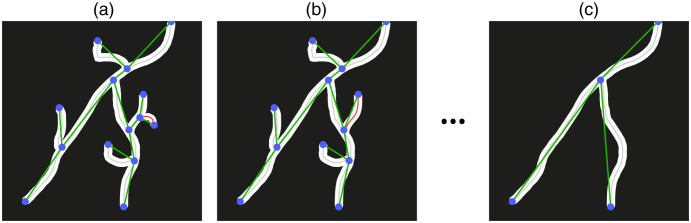
Illustration of the pruning procedure. (a) The smallest branch (red) is detected. (b) The removal of the smallest branch generates a new branch, which is also removed because its arc-length is smaller than a threshold size. The branches are removed until there are no branches smaller than the threshold. The final result is shown in (c).

The graph, or equivalently, the skeleton, is then pruned so that small branches with lengths smaller than a fixed value S are removed. A branch is defined as an edge connecting a terminal and a bifurcation node. The size of a branch is calculated as the arc-length of the pixels representing the branch. The pruning procedure proceeds iteratively. As illustrated in Fig. 6, the smallest branch in the whole graph is removed first, which defines a new graph that does not contain the removed branch. Note that the removal of a branch might generate a new branch in the graph, in which case the length of the new branch is calculated and stored. Then, the smallest branch in the new graph is removed, and it is verified if the removal generated a new branch. The procedure is repeated until there are no more branches smaller than S.

Although the pruning procedure might lead to the removal of genuine blood vessel segments, it usually allows for a large reduction in false positive branches. Still, it is important to select a proper value of S to avoid excessive pruning. The value of S should be slightly larger than the typical diameter of a vessel. This allows for the removal of small branches generated from inaccuracies on the segmentation of the vessels’ walls.

For 2D samples, the generated graph has the disadvantage that two blood vessels crossing each other may be detected as a branching point. Pattern recognition and machine learning approaches can be used for differentiating between crossings and bifurcations.^18^^,^^27^^–^^29^ But this is still an active area of research. Therefore, detecting spurious bifurcation points in 2D samples should be expected.

### 3.4 Morphological Measurements

Having obtained the skeleton and the graph, many morphological measurements can be calculated for characterizing the vascular network. We focus on calculating the vessel density, density of bifurcation points, and vessel tortuosity.

#### 3.4.1 Vessel density

The vessel density is defined as the sum of lengths of all blood vessel segments divided by the image volume. A vessel segment is composed of the set of pixels that connects two bifurcation points or a termination and a bifurcation point. Formally, with P being the set of blood vessel segments of an image, al(P[i]) being the arc-length of the i’th blood vessel segment, and V being the area or volume of the image, the vessel density (VD) is calculated as

$$\mathrm{VD}=\frac{\underset{i}{\sum }al(P[i])}{V}.$$

Typically, the physical units are defined in millimeters. Therefore, the vessel density is commonly written as $\mathrm{mm}/{\mathrm{mm}}^{2}$ for 2D images and $\mathrm{mm}/{\mathrm{mm}}^{3}$ for 3D images.

#### 3.4.2 Vessel branching

The density of bifurcation points is defined as the number of bifurcation points, identified as nodes in the graph having degree equal to or larger than 3, divided by the area (for 2D samples) or by the volume (for 3D samples) of the sample.

#### 3.4.3 Vessel tortuosity

**Fig. 7:**
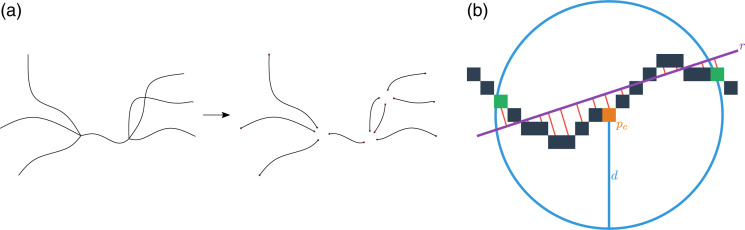
Illustration of the methodology for calculating blood vessel tortuosity. (a) The skeleton of the vascular network is divided into segments. (b) The tortuosity at a reference pixel $p_{c}$ (in orange) is calculated as the average length of the red lines shown in the figure, which represent the smallest distances between the pixels and line r (in purple).

The blood vessel tortuosity is calculated for each pixel of all blood vessel segments [Fig. 7(a) shows examples of segments). The calculation of the tortuosity for a particular pixel of a vessel segment is illustrated in Fig. 7(b). For a given reference pixel $p_{c}$ [shown in orange in Fig. 7(b)], its local neighborhood is composed of all pixels inside a circle of radius d centered at $p_{c}$. A line r is adjusted to the local neighborhood using linear least-squares regression. The tortuosity value assigned to $p_{c}$ is calculated as the average of the smallest distances between each pixel in the local neighborhood and line r. The parameter d adjusts the scale of the calculated tortuosity, which allows for the detection of different types of tortuous structures. Small values of d can be used for detecting sharp turns of the blood vessels, whereas large values of d lead to the identification of smooth changes in direction of the vasculature. The overall tortuosity of the vascular network in an image is calculated as the average tortuosity obtained for the considered reference pixels.

## 4 Case Examples

In the following, examples of 2D and 3D vascularity characterization using Pyvane are presented. The blood vessels contained in the considered samples are automatically identified, and the vascular density, number of branching points, and tortuosity are calculated.

### 4.1 2D Analysis

**Fig. 8:**
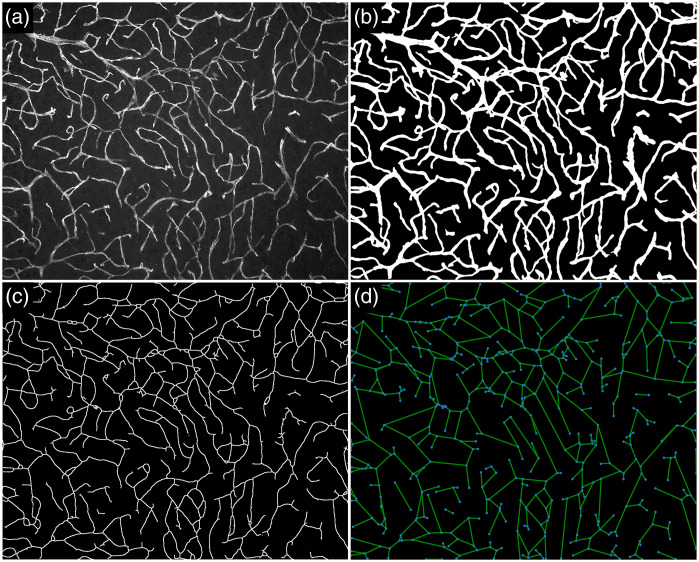
Example of application of the method to one of the samples. (a) Original sample. (b) Detected vascular network, represented as a binary image. (c) Skeleton of the vascular network. (d) Graph representing bifurcations and terminations (shown in blue), as well as their connections (shown in green).

Regarding 2D images, Fig. 8 shows an example of intermediate results obtained for one of the samples. The original sample is shown in Fig. 8(a), and the respective result of the vasculature detection is shown in Fig. 8(b). The skeleton of the detected vasculature is shown in Fig. 8(c). The skeleton provides a representation of the vasculature in the original sample and can be used for measuring some morphological properties such as length and tortuosity. Figure 8(d) shows the representation of the vasculature as a graph. The graph provides a concise description of the topology of the vascular network and can be used for calculating bifurcation and branch statistics.

**Fig. 9:**
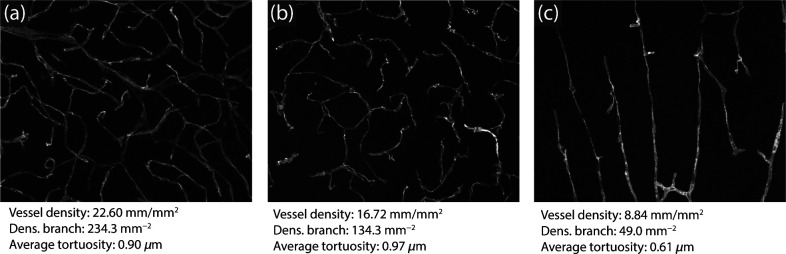
Measurements obtained for three selected 2D samples. Sample (a) has the largest density among the selected samples, whereas (b) has the lowest density and the largest average tortuosity. Sample (c) has low average tortuosity.

Figure 9 shows examples of samples and the respective measurements that were obtained. We chose samples with clearly distinct properties for visual interpretation. Samples having more subtle differences can also be detected and quantified using the methodology. This is important as Pyvane allows for the identification of small changes in morphology due to experimental conditions, which may not be detected by visual inspection.

### 4.2 3D Analysis

**Fig. 10:**
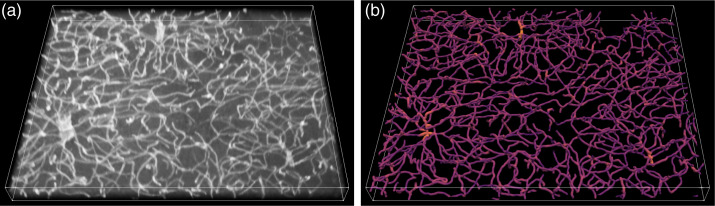
Example of 3D vascular network detection and digital reconstruction. (a) Original sample. (b) Visualization of the detected blood vessels. The colors indicate the diameter of the blood vessels, with brighter colors representing thicker vessels.

**Fig. 11:**
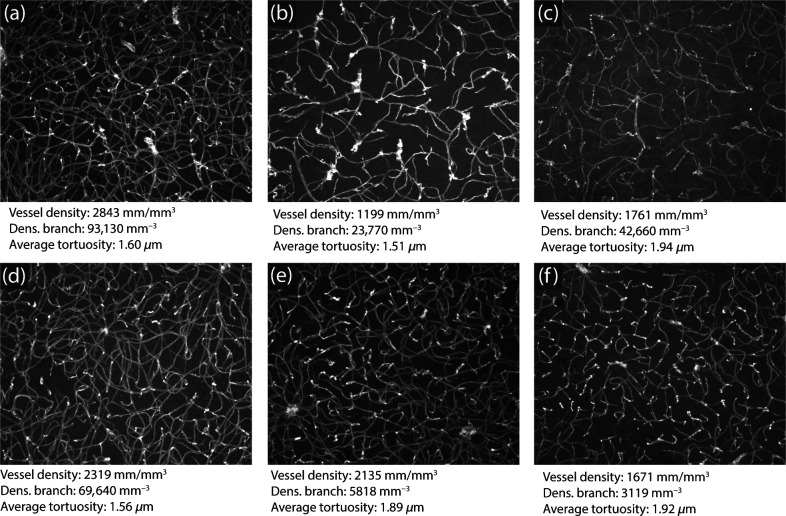
Measurements obtained for six selected 3D samples. Sample (a) has the largest density among the selected samples, whereas (b) has the lowest density. The vasculature in (c) has large average tortuosity, which is a consequence of its irregular blood vessel segments. Sample (d) has relatively low average tortuosity. Samples (e) and (f) have intermediate values of the considered measurements. The images shown are maximum Z-projections of the original samples.

Regarding 3D samples, Fig. 10 shows an example of a 3D stack, the respective reconstruction of the vasculature using the identified blood vessels, and an estimation for the diameter of each blood vessel. Figure 11 shows examples of measurements obtained regarding the morphology of 3D vascular networks. Having the vasculature represented as a skeleton and a graph, the considered properties can be easily measured and provide a reliable characterization of the samples.

**Fig. 12:**
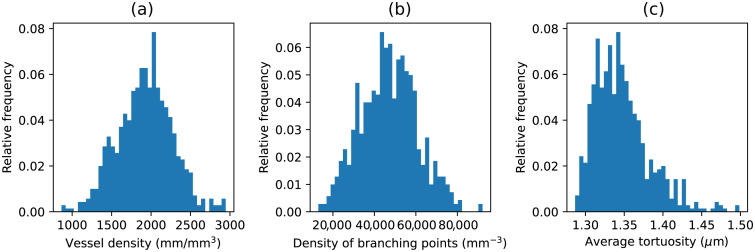
Distribution of the (a) vessel density, (b) density of branching points, and (c) average tortuosity for the considered set of 3D samples.

To illustrate the potential of the immunofluorescent labeling protocol and Pyvane, the vessel and branching point density as well as the average tortuosity were measured for a set of 324 3D stacks. Figure 12 shows the distributions of the obtained measurements. The distribution of the vessel and branching density are symmetric and peak at around $2000\;\mathrm{mm}/mm^{3}$ and $50,000\;{\mathrm{mm}}^{-3}$, respectively. Regarding the average tortuosity, it can be observed that the vasculature of a small set of samples is more tortuous than the majority of the samples, leading to a skewed distribution.

**Fig. 13:**
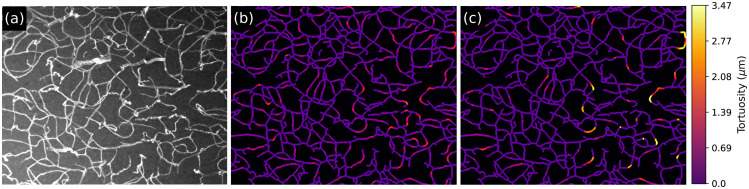
Tortuosity values calculated for individual pixels of blood vessels. The tortuosity of the vasculature shown in (a) was calculated at a scale of (b) d=10  μm and (c) d=20  μm.

Regarding the tortuosity of blood vessels, it can also be investigated locally. As presented in Sec. 3.4.3, each pixel in the vasculature can have an associated tortuosity value. Figure 13 shows an example of an original sample [Fig. 13(a)] and the respective local tortuosity values at a small scale [Fig. 13(b)] and at a large [Fig. 13(c)] scale. For the small scale, sharp changes in direction are detected. If a large scale is considered, segments of the blood vessels with smooth but prolonged changes in direction are identified. This local tortuosity can be used for detecting possible restrictions of blood flow^30^^,^^31^ and for characterizing angiogenesis.^32^^–^^34^

Indeed, the spatial scale constitutes an important point to be taken into account while performing the described operations as it can influence the results. The definition of the proper scale of analysis should consider not only the scale of noise and other eventual objects to be removed but also the spatial extensions involved in the biological phenomenon being studied.

It is also interesting to investigate the influence of using 2D or 3D data when estimating the tortuosity. To do so, 2D samples were generated by discarding the depth information from the graphs generated from the 3D samples. As expected, when the tortuosity is calculated in 2D, the obtained values tend to be smaller than in 3D because variations along the depth of the sample are not considered. For instance, the average tortuosity of all 2D samples analyzed was 0.93 (0.07), whereas the average obtained for the 3D samples was 1.4 (0.1). The values between parentheses indicate standard deviations.

## 5 Conclusion

This protocol presents a straightforward procedure for immunostaining of brain vasculature followed by unbiased structural quantifications. The examples provided here demonstrate how vessel density, branching, and tortuosity can be obtained from 2D or 3D microscopic images. As processing power continues to grow, the ability to reliably quantify detailed blood vessel morphology, independent of human biases, is becoming more accessible. The goal of this protocol is to provide the community with tools that can be easily applied to promote more consistency in vascular data analysis.
